# Bills of Mortality, for Portsmouth, Newhampshire, for 1805, 1806, 1807, 1808

**Published:** 1809-06

**Authors:** Lyman Spalding


### BILLS OF MORTALITY,

*For Portsmouth, Newhampshire, for* 1805, 1806, 1807, 1808.

By LYMAN SPALDING, M. D.

| | | | |
|---|---|---|---|
| Abscess | 1 | Hooping Cough | 4 |
| Angina Pectoris | 1 | Intemperance | 1 |
| Aphtha | 2 | Jaundice | 2 |
| Apoplexy | 1 | Mortification | 3 |
| Asphyxia | 1 | Old Age | 6 |
| Atrophy | 3 | Palsy | 6 |
| Bedridden | 1 | Quinsy | 9 |
| Bleeding | 1 | Small-Pox, natural | 1 |
| Bleeding at the Lungs | 1 | Strangury | 1 |
| Cholera of Infants | 12 | Sudden | 4 |
| Consumption | 30 | CASUALTIES. | |
| Convulsions | 10 | Drowned | 3 |
| Croup | 7 | Fall | 2 |
| Dropsy | 5 | Frozen | 1 |
| Dropsy in the Brain | 4 | Suicide | 1 |
| Dropsy in the Chest | 1 | BIRTHS. | |
| Erythema | 1 | Males 138, Females 157 | 295 |
| Fever Puerperal | 1 | | |
| Fever Pulmonic | 6 | MARRIAGES | 67 |
| Herpes | 1 | | |

PORTSMOUTH,

Portsmouth, the capital of the state of Newhampshire, situated 43° 5′ north latitude, and 6° 26′ east longitude from Washington, contains above 6500 inhabitants.

The death by asphyxia was by attending a crowded evening lecture. Bedridden, this old lady had been confined to her bed more than sixteen years. The consumption has made its usual ravages.

1806.

| | |
|---|---|
| Aphtha | 5 |
| Apoplexy | 2 |
| Atrophy | 13 |
| Bedridden | 1 |
| Bleeding at the Lungs | 1 |
| Cancer | 2 |
| Cholera of Infants | 6 |
| Consumption | 21 |
| Convulsions | 7 |
| Croup | 1 |
| Dropsy | 5 |
| Dropsy in the Brain | 3 |
| Dropsy on the Brain | 1 |
| Dysentery | 1 |
| Erythema | 1 |
| Fever from Worms | 1 |
| Fever Pulmonic | 6 |
| Fever Typhus | 4 |
| Gout | 1 |
| Herpes | 1 |
| Hooping Cough | 1 |
| Insanity | 3 |
| Inflammation of the Stomach | 1 |
| Mortification | 4 |
| Nephritis | 1 |
| Old Age | 9 |
| Palsy | 2 |
| Phrenitis | 2 |
| Quinsy | 4 |
| Spina Bifida | 1 |
| Scrophula | 1 |
| Strangury | 1 |
| Tetanus | 1 |
| Ulcer carious | 1 |
| **CASUALTIES.** | |
| Burnt | 1 |
| Drowned | 1 |
| Poisoned by Opium | 2 |
| **BIRTHS.** | |
| Males 128, Females 128 | 256 |
| Marriages | 63 |

1807.

| | |
|---|---|
| Aphtha | 1 |
| Aneurism | 1 |
| Asthma | 2 |
| Atrophy | 12 |
| Bleeding at the Umbilicus | 1 |
| Cholera of Infants | 1 |
| Consumption | 30 |
| Convulsions | 6 |
| Dropsy | 5 |
| Dropsy in the Brain | 2 |
| Drunkenness | 1 |
| Epilepsy | 1 |
| Erythema | 1 |
| Fever bilious | 3 |
| Fever from Worms | 2 |
| Fever Inflammatory | 1 |
| Fever Intermittent | 1 |
| Fever Typhus | 1 |
| Fever Pulmonic | 5 |
| Herpes | 1 |
| Influenza | 7 |
| Jaundice | 1 |
| Mortification | 7 |
| Old Age | 10 |
| Palsy | 5 |
| Quinsy | 1 |
| Schirrus Liver | 1 |

Sudden

| | | | |
|---|---|---|---|
| Sudden | 4 | BIRTHS. | |
| CASUALTIES. | | Males 151, Females 138 | 284 |
| Drowned | 4 | MARRIAGES | 62 |
| Scalded | 1 | | |

On the 23d of January, at sunrise, the thermometer was 10 deg. on the 26th 13 deg. below Zero; the coldest weather ever recorded in this town. By 8 o'clock on those days the town was so completely filled with an intensely thick fog or vapour from the Piscataqua river, as to render it almost impossible to see across the streets. A severe rain storm immediately followed each of those days. Between the 23d and 26th the Influenza made its appearance, and after visiting almost every family in town, subsided early in May.

About the middle of August the Influenza made its second appearance, which was more severe than the first, sparing none, not even those who suffered the most in the former attack. After destroying a few and predisposing many to Consumption, it disappeared in December. The mild Typhus fever appeared in October, and prevailed through the year.

1808.

| | | | |
|---|---|---|---|
| Abscess | 1 | Mortification | 3 |
| Angina Pectoris | 1 | Nephritis | 1 |
| Aphtha | 4 | Nonclosure of the Foramen Ovale and Canalis Arteriosus | 1 |
| Apoplexy | 1 | Old Age | 7 |
| Atrophy | 8 | Palsy | 1 |
| Bleeding at the Stomach | 1 | Phrenitis | 1 |
| Cholera of Infants | 3 | Schirrous Bladder | 1 |
| Consumption | 20 | Schirrous Liver | 2 |
| Convulsions | 12 | Sudden | 3 |
| Croup | 1 | Worms | 1 |
| Dropsy | 4 | CASUALTIES. | |
| Dropsy in the Brain | 4 | Burnt | 1 |
| Dysentery | 2 | Drowned | 4 |
| Erythema | 2 | Suicide | 1 |
| Fever Bilious | 3 | BIRTHS. | |
| Fever Puerperal | 1 | Males 141, Females 134 | 275 |
| Fever Pulmonic | 5 | Still Born | 8 |
| Fever Typhus | 1 | MARRIAGES | 56 |
| Hooping Cough | 5 | | |
| Intoxication | 1 | | |
| Malignant Sore Throat | 1 | | |

INTELLIGENCE.

